# Effect of high‐speed exercise on subchondral bone in the metacarpo−/metatarsophalangeal joints of 2‐year‐old Thoroughbred racehorses in their first year of training

**DOI:** 10.1111/evj.14524

**Published:** 2025-05-05

**Authors:** Sarah A. Ciamillo, Kathryn W. Bills, Taryn M. Gassert, Dean W. Richardson, Kara A. Brown, Darko Stefanovski, Kyla F. Ortved

**Affiliations:** ^1^ Department of Clinical Studies, New Bolton Center University of Pennsylvania Kennett Square Pennsylvania USA; ^2^ Sports Medicine Associates of Chester County Cochranville Pennsylvania USA

**Keywords:** furlong, high‐speed exercise, horse, injury prevention, subchondral bone pathology

## Abstract

**Background:**

Stress‐induced bone injury can lead to catastrophic injuries in Thoroughbred racehorses. Accumulation of high‐speed exercise can increase the risk of subchondral bone injury.

**Objectives:**

To investigate the effect of high‐speed exercise on the subchondral bone of the metacarpo–metatarsophalangeal (MCP/MTP) joints using standing cone‐beam computed tomography (CBCT) in young racing Thoroughbreds.

**Study Design:**

Observational cohort study.

**Methods:**

Forty‐one 2‐year‐old Thoroughbred racehorses during their first year of training were evaluated at 0, 6 and 12 months. Horses were evaluated for lameness and effusion of the MCP/MTP joints, and then standing CBCT was performed of the MCP/MTP joints. Subchondral bone hyperdensity (sclerosis) was measured (mm) at defined locations in the distal aspect of the third metacarpal (MC3)/metatarsal (MT3) bone and proximal phalanx (P1). CBCT scans were evaluated for subchondral bone hypodensity (pathology) in MC3/MT3, P1 and proximal sesamoid bones. Racing and training records were obtained from a national online database and trainers.

**Results:**

Subchondral bone sclerosis (coefficient = 0.45; *p* < 0.003; 95% confidence interval [CI] 0.16–0.74) and pathology (IRR = 1.01; *p* < 0.001; 95% CI 1.00–1.01) increased significantly with the number of high‐speed furlongs accumulated. Lameness was not associated with sclerosis or pathology; however, joint effusion was associated with both sclerosis (IRR = 1.01; *p* = 0.02; 95% CI 1.00–1.02) and pathology lesion (IRR = 1.14; *p* < 0.01; 95% CI 1.04–1.25).

**Main Limitations:**

Limitations include attrition of horses over time and lack of control over training and husbandry.

**Conclusions:**

An increase in high‐speed work was associated with both an increase in subchondral bone sclerosis and pathology.

## 1 INTRODUCTION

Musculoskeletal injuries are the most common cause of lost training days and racing‐associated death in Thoroughbred racehorses.^1^, ^2^, ^3^ During race training, bone experiences high strain proportional to speed in a repetitive manner that can lead to microdamage and fatigue over time.^4^ Several studies have demonstrated an association between high‐speed work and stress fractures in Thoroughbred racehorses.^5^, ^6^, ^7^, ^8^, ^9^ Understanding this association is critical to limiting training‐associated injuries and catastrophic failures through early identification of lesions and adaptations to training. Until recently, subtle changes in bone morphology were difficult to diagnose with conventional imaging tools such as radiography and ultrasonography. Cross‐sectional imaging modalities, including computed tomography (CT), allow for a more detailed examination of bone structure and are being used with increasing frequency to detect early development of pathology and serially evaluate horses over time.^10^, ^11^, ^12^, ^13^, ^14^

High‐speed work is necessary to induce bone adaptations that are protective against damage,^15^ however, an overabundance of cumulative high‐speed work is associated with fatigue damage and increased risk of fractures.^7^ Previous studies have demonstrated that more cumulative racing and high‐speed training in the previous season carry an increased risk of subchondral bone injury of the metacarpal/metatarsal condyles^16^ and fatigue fracture of the proximal sesamoid bones.^9^ Horses <3 years of age have been reported to be at increased risk of fracture, which may be due to inadequate/incomplete adaptation of the material and structural properties of long bones.^17^ Despite the association between high‐speed exercise and bone fatigue injury in horses, the relationship is clearly complex and training regimens proven to optimise bone strength while minimising injury remain elusive.

Recently, changes in subchondral bone morphology and development of pathology in metacarpophalangeal (MCP) and metatarsophalangeal (MTP) joints of young Thoroughbred racehorses imaged using standing cone beam computed tomography (CBCT) over a 12‐month period of race training were described.^11^ This study showed that subchondral bone pathology increased over time in the medial and lateral palmar/plantar condyles of the third metacarpal bone (MC3) and third metatarsal bone (MT3), the lateral parasagittal groove of MC3/MT3, the medial dorsal condyle of MC3/MT3, and the medial and lateral eminences of the proximal phalanx (P1). We also found that subchondral bone hyperdensity or sclerosis increased significantly over time in the medial and lateral palmar/plantar condyles of MC3/MT3 and the medial and lateral parasagittal grooves of MC3/MT3.

Although our first study demonstrated clear changes in the MCP/MTP joints over time, the association between high‐speed training and the development of pathology in this group of horses remains unknown. Therefore, the primary objective of this study was to investigate the effect of high‐speed work, as estimated by high‐speed furlongs (HSF) recorded, on subchondral bone morphology and pathology of the MCP/MTP joints using standing CBCT in young racing Thoroughbreds. The secondary objective of this study was to investigate the association between subchondral bone morphology and pathology with clinical parameters including lameness and joint effusion. We hypothesised that (1) subchondral bone sclerosis would increase in the MCP/MTP joints with an increased number of HSFs; (2) the prevalence of subchondral bone pathology would increase in the MCP/MTP joints with an increased number of HSFs; and (3) lameness and MCP/MTP joint effusion would both be associated with subchondral bone sclerosis and pathology.

## 2 MATERIALS AND METHODS

### 2.1 Animals

Forty‐one 2‐year‐old Thoroughbred racehorses in their first year of race training were enrolled and were evaluated at 0, 6 and 12 months into high‐speed race training. At all evaluations, horses underwent a general physical examination and lameness examination by two board‐certified equine sports medicine and rehabilitation specialists (KFO and KAB) with the average score of the two observers used in the final data analysis. Horses were evaluated at the trot in a straight line on asphalt, and lameness was graded using the AAEP lameness scale.^18^ All MCP/MTP joints were palpated and graded for degree of joint effusion using a subjective grading system (0 = none; 1 = mild; 2 = moderate; 3 = severe). For the duration of the study, horses trained and raced as dictated by the individual trainer. Medical history, including injuries and periods of rest, was obtained from trainers and referring veterinarians. Exercise history, including official timed high‐speed works and races, was obtained from an online national database of racing information and statistics (Equibase Company LLC) and from individual horse trainers and managers. Total HSF (including during racing and official timed works) during the study period, total number of official high‐speed works, average speed per HSF official high‐speed works during the study period, and rest weeks in which trainers or managers refrained from training the horse were collected for each horse.

### 2.2 Standing cone beam computed tomography

Standing CBCT scans of the MCP/MTP joints were obtained as previously described.^11^ Briefly, horses were sedated with an alpha‐2 agonist (xylazine; Rompun®, Bayer or detomidine; Dormosedan®, Zoetis) +/− morphine sulfate (Hospira, Inc.) administered intravenously (IV). To facilitate camera detection and motion correction, a heat‐moulded polymer cuff (acrylonitrile butadiene styrene [ABS] plastic) with 5 arms and peripheral reflective markers was then attached to the lateral aspect of the distal limb and secured with nonreflective cloth tape (Pro Gaff by Pro Tapes). Using a synchronised x‐ray tube (VarexB147H housing/G892 insert, Varex Imaging) and detector (Varian 4343CB Varex Imaging) mounted to articulated manufacturing robots (IRB6700, ABB), diverging x‐rays of 15 frames/second for a total of 30 seconds/scan and 451 images were generated during a single 190‐degree circular path. Following acquisition, images were reconstructed using Adaptive Contrast Enhancement software (Version 1.1.5.0; Kromtech) to 0.5 mm thickness and corrected for motion in a 512 × 512 reconstruction matrix (0.5 mm isotropic voxel). The final images were randomised and blinded by a third party (SAC) and evaluated in DICOM viewing software (OsiriX MD 11.0). CBCT scans were blindly evaluated for subchondral bone hyperdensity (sclerosis) and subchondral bone hypodensity (pathology) in distal MC3/MT3, proximal P1 and the proximal sesamoid bones.

### 2.3 Subchondral bone hyperdensity (sclerosis)

**FIGURE 1:**
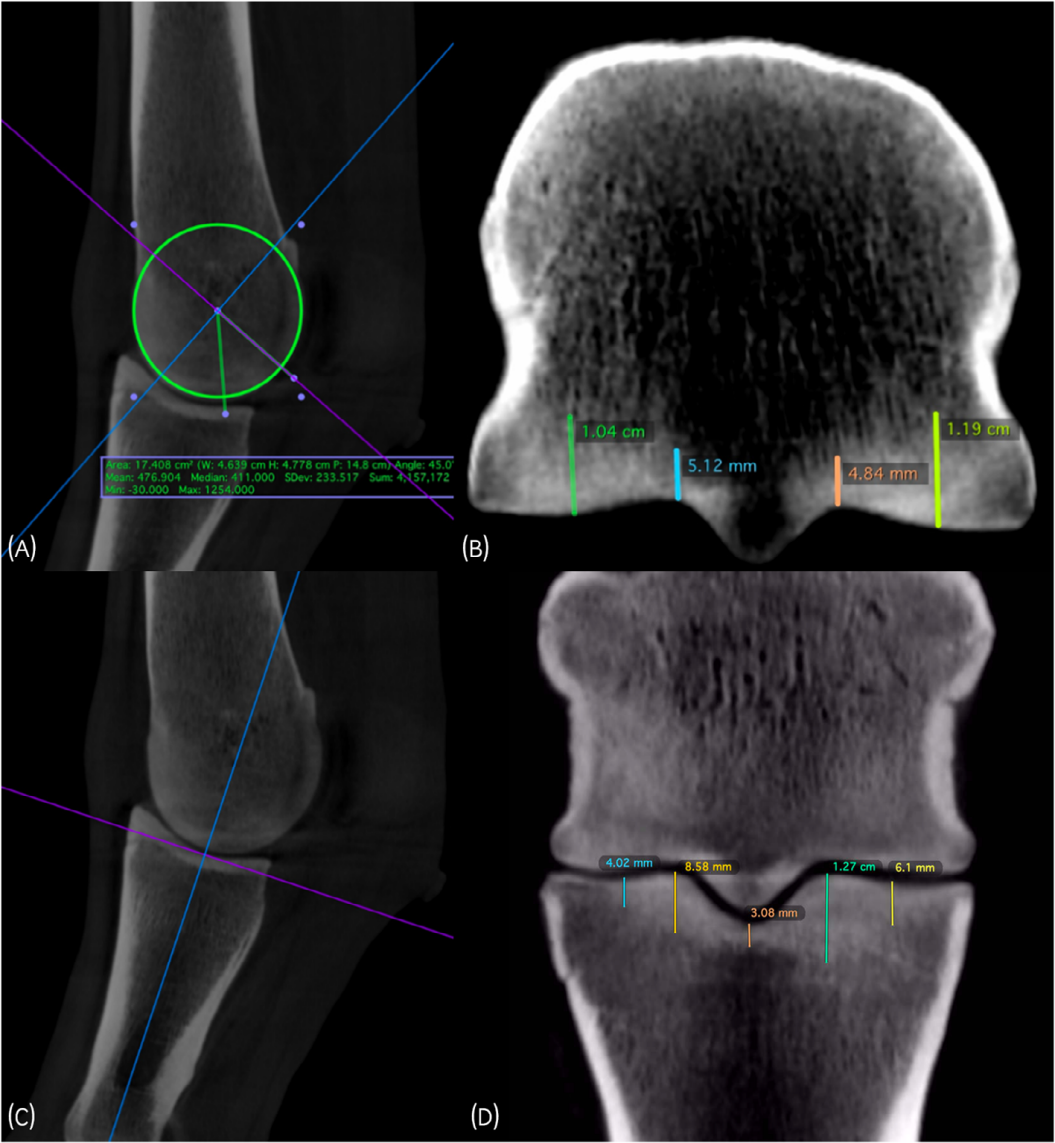
(A) Standard plane for measurement of metacarpal (MC)3/metatarsal (MT)3 hyperdensity (sclerosis) in the (B) medial condyle, medial parasagittal groove, lateral parasagittal groove and lateral condyle (left to right). (C) Standard plane for measurement of proximal phalanx (P1) hyperdensity (sclerosis) in the (D) medial fovea, medial ridge of P1, sagittal groove, lateral ridge of P1 and lateral fovea (left to right).

As previously described, MC3/MT3 and P1 subchondral hyperdensity (sclerosis) was measured (mm) using a single, reformatted image by a single, blinded reviewer (SAC).^11^ OsiriX software (OsiriX MD 11.0) was used to generate images and to measure the extent of trabecular hyperdensity (Figure 1). Briefly, for MC3/MT3, a single, dorsoproximal to palmarodistal oblique plane was generated, oriented at 45 degrees from the longitudinal axis of the diaphysis that intersected the palmar/plantar aspect of the condyle along a radian from the centre of the condyle. The height (mm) of hyperdensity in the subchondral bone was measured at 4 sites including: (1) medial mid‐condyle (midway between the most abaxial portion of the condyle to the medial parasagittal groove), (2) medial parasagittal groove, (3) lateral parasagittal groove and (4) lateral mid‐condyle (midway between the most abaxial portion of the condyle to the lateral parasagittal groove). For P1, a single dorsal plane image was acquired 10 mm dorsal to a plane drawn midway between the medial and lateral palmar/plantar eminences and perpendicular to a line between the dorsal and palmar aspects of the proximal articular margin of P1. The height (mm) of hyperdensity in the subchondral bone was measured at 5 sites including: (1) medial mid‐fovea, (2) lateral mid‐fovea, (3) medial eminence of P1, (4) lateral eminence of P1 and (5) the sagittal groove. The reviewer was able to manipulate the window width and level of the images. Hyperdensity was not measured in the proximal sesamoid bones. The average of the sum of sclerosis in MC3/MT3 and P1 in all MCP/MTP joints was calculated to provide total sclerosis per horse.

### 2.4 Subchondral bone hypodensity (pathology)

Once all CBCT scans were acquired for all horses at all time points, the scans were analysed by a board‐certified equine surgeon (KFO) and board‐certified equine radiologist (KWB). Multiplanar reconstructions were evaluated for the presence of subchondral hypodensities surrounded by regions of hyperdensity consistent with subchondral demineralisation (pathology) as previously described.^10^, ^11^ Thirteen specific locations were evaluated for the presence (yes or no) of subchondral bone pathology: medial and lateral palmar and plantar MC3/MT3 condyles, medial and lateral dorsal and supracondylar aspects of the MC3/MT3 condyles (dorsal MC3/MT3 condyles), medial and lateral parasagittal grooves of MC3/MT3, medial and lateral ridge of P1, medial and lateral fovea of P1, sagittal groove of P1 and medial and lateral dorsal proximal sesamoid bones, as previously described.^11^ A total subchondral bone lesion number per horse (all lesions in all locations in all MCP/MTP joints) was calculated at each time point.

### 2.5 Data analysis

Following complete examination of all CBCTs, the studies were unblinded to determine the time point the images were obtained at and the corresponding limb and horse. All data was analysed using Stata 17MP (StataCorp LLC) with two‐sided tests of hypotheses and a *p* value <0.05 as the criterion for statistical significance. Tests of normal distribution (Shapiro–Wilk test) were performed to determine the extent of skewness of the data. Descriptive analyses included computation of medians, interquartile ranges (IQR) of continuous variables, and tabulation of categorical variables.

For the purpose of inference statistics, two types of multilevel mixed‐effects (linear and Poisson) regression models depending on the distribution of the outcomes (total subchondral bone sclerosis and total subchondral bone lesions) were used to examine the association with the fixed effects, including the number of HSF and the number of high‐speed works. Because there were repeated measures in one animal over time, time in months was used as a confounder. These models were also used, depending on the distribution of the outcomes, to examine the association between clinical parameters (lameness and joint effusion) and subchondral bone sclerosis and subchondral bone lesions. To account for repeated measures in one animal over time, time in months was used as a confounder. Random effects were set at the level of the individual animal. The results are reported as incidence rate ratio (IRR) or coefficient with their respective 95% confidence interval (CI) and *p* value. *p* value <0.05 was considered to be significant. For illustration purposes, Spearman's rank correlation was used to plot the association between HSF and total subchondral bone sclerosis and HSF and total subchondral bone lesions.

## 3 RESULTS

### 3.1 Descriptive statistics

**FIGURE 2:**
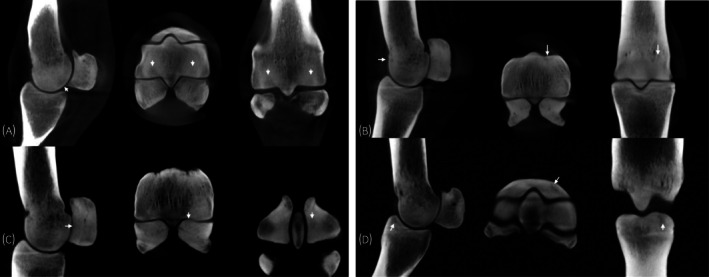
Representative sagittal (left), transverse (middle) and dorsal (right) plane images of subchondral bone pathology (arrows) observed in certain anatomic locations of the metacarpophalngeal (MCP)/metatarsophalanglea (MTP) joint. Lateral is to the left and medial is to the right in all images. (A) Subchondral bone pathology in the lateral and medial palmar condyles of MC3. (B) Subchondral bone pathology in the medial dorsal condyle of MC3. (C) Subchondral bone pathology in the dorsal medial proximal sesamoid bone. (D) Subchondral bone pathology in the medial fovea of P1.

Forty‐one Thoroughbred racehorses, including 16 males, 23 females and 2 geldings, were enrolled in the study at the beginning of their 2‐year‐old year in training. All 41 horses were evaluated at 0 months, while 30 horses were evaluated at 6 months and 34 horses were evaluated at 12 months. Nineteen horses were evaluated at all 3 time points. There were 7 trainers associated with the 41 horses throughout the study. One trainer had 16 horses, one trainer had 8 horses, one trainer had 7 horses, one trainer had 6 horses, one trainer had 2 horses and two trainers each had 1 horse. A total of 364 CBCT scans were reviewed. The complete details of subchondral bone morphology and pathology over time in this cohort of horses have been previously described.^11^ A summary of total subchondral bone sclerosis (mm) measured in MC3/MT3 and P1 and total subchondral bone lesions noted in the MCP/MTP joints is shown in Table 1. Representative images of subchondral bone injury in some locations are shown in Figure 2. Total subchondral bone sclerosis increased significantly over time. Total subchondral bone sclerosis increased significantly between 0 and 12 months (*p* < 0.001) and 6 and 12 months (*p* = 0.032); however, the increase in subchondral bone sclerosis between 0 and 6 months was not significant (*p* = 0.53). The total number of subchondral bone lesions was significantly increased between 0 to 6 months (*p* < 0.001), 0 and 12 months (*p* < 0.001) and 6 and 12 months (*p* = 0.032). Descriptive statistics of lameness scores, joint effusion scores, subchondral bone sclerosis and the number of subchondral bone lesions in each MCP/MTP joint at 0, 6 and 12 months are shown in Table 2.

**TABLE 1:** Descriptive statistics including median (range) of computed tomographic (CT) findings and exercise characteristics.

	0 months	6 months	12 months
Subchondral bone sclerosis (mm)	210.92 (131.53–252.46)	244.89 (146.09–319.3)	271.39 (197.2–350.41)
Subchondral bone lesions	3 (0–13)	6.5 (1–19)	8.5 (1–25)
Total HSF	0 (0–10)	17 (0–85)	10 (0–148.50)
Total high‐speed works	0 (0–3)	5 (0–18)	5.5 (0–27)
Average speed per HSF (s)	13.10 (12.99–13.2)	12.59 (12.21–12.93)	12.59 (12.0–12.91)
Time off (weeks)	0 (0–0)	3.75 (0–24)	1 (0–20)

Abbreviation: HSF, high‐speed furlong.

**TABLE 2:** Descriptive statistics including median (range) of lameness and joint effusion scores, and computed tomographic (CT) findings.

Limb	Variable	0 months	6 months	12 months
Left front	Lameness score	0 (0–1)	0 (0–1.5)	0.5 (0–2)
	Joint effusion score	0 (0–1)	0.5 (0–1)	0.5 (0–1)
	Subchondral bone sclerosis (mm)	52.75 (19.84–81.73)	59.52 (27.52–84.65)	63.47 (45.99–99.73)
	Subchondral bone lesions	1 (0–4)	1.5 (0–6)	2 (0–7)
Right front	Lameness score	0 (0–1.5)	0.5 (0–1.5)	0.5 (0–2)
	Joint effusion score	0 (0–1.5)	0 (0–1)	0.5 (0–1)
	Subchondral bone sclerosis (mm)	54.14 (27.95–69.23)	56.12 (30.66–107.43)	68.71 (41.09–93.80)
	Subchondral bone lesions	1 (0–4)	2 (0–6)	2.5 (0–6)
Left hind	Lameness score	0 (0–1)	0 (0–1.5)	0 (0–1.5)
	Joint effusion score	0 (0–1.5)	0.5 (0–1)	0.5 (0–1)
	Subchondral bone sclerosis (mm)	52.11 (34.37–77.40)	63.73 (42.43–95.41)	68.73 (52.13–108.05)
	Subchondral bone lesions	1 (0–6)	2 (0–6)	2 (0–6)
Right hind	Lameness score	0 (0–1.5)	0.5 (0–1.5)	0.5 (0–1)
	Joint effusion score	0 (0–1.5)	0.5 (0–1)	0.5 (0–1)
	Subchondral bone sclerosis (mm)	52.33 (35.95–71.57)	53.18 (36.72–90.1)	56.33 (39.99–81.02)
	Subchondral bone lesions	0 (0–4)	2 (0–6)	2 (0–6)

### 3.2 Effect of high‐speed exercise

**FIGURE 3:**
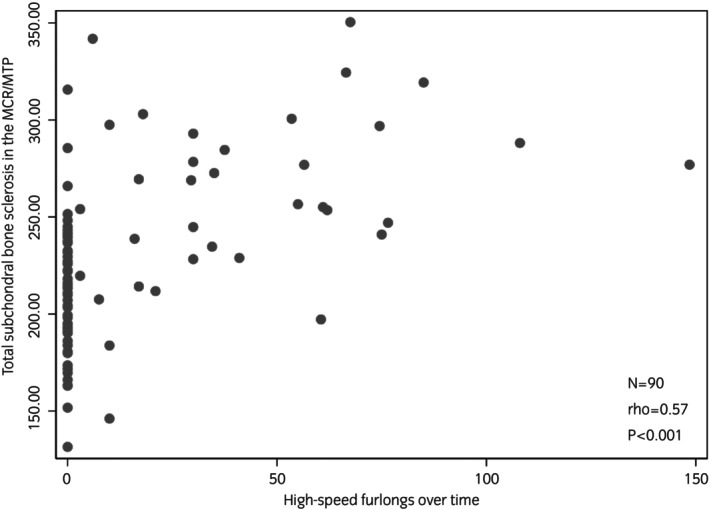
Association between total subchondral bone sclerosis in the metacarpophalangeal (MCP)/metatarsophalangeal (MTP) joints of Thoroughbred racehorses and high‐speed furlongs over time assessed using Spearman's rank correlation.

When adjusted for time, there was a significant association between the total number of HSF recorded and total sclerosis of the MC3/MT3 condyles and P1 (coefficient = 0.45; *p* < 0.003; 95% CI 0.16–0.74). Graphical representation of this association is shown in Figure 3. For every 1 HSF accumulated, there was a 0.5 mm increase in total sclerosis. There was also a significant association between the total number of high‐speed works recorded and total sclerosis of the MC3/MT3 condyles and P1 (coefficient = 2.34; *p* < 0.001; 95% CI 0.91–3.77). For every high‐speed work accumulated, there was a 2.3 mm increase in total sclerosis. When adjusted for time, there was not a significant association between the average speed per HSF and total sclerosis (coefficient = −36.22; *p* = 0.3; 95% CI −101.49 to 29.05). Additionally, there was not a significant association between the amount of time off a horse had and total sclerosis (coefficient = 0.29; *p* = 0.8; 95% CI −2.12 to 2.71).

**FIGURE 4:**
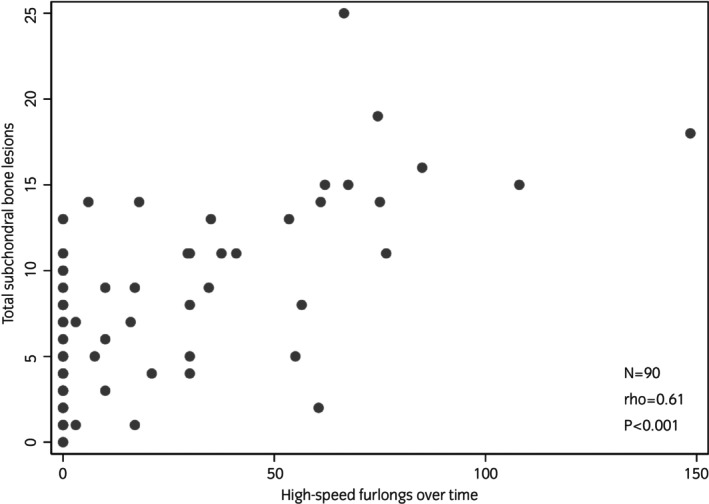
Association between total subchondral bone lesions in the metacarpophalangeal (MCP)/metatarsophalangeal (MTP) joints of Thoroughbred racehorses and high‐speed furlongs over time assessed using Spearman's rank correlation.

When adjusted for time, there was a significant association between the total number of HSF recorded and the total number of lesions identified in the MCP/MTP subchondral bone of horses (*p* < 0.001). Graphical representation of this association is shown in Figure 4. The incidence rate ratio (IRR), which represents the change in risk for every 1 HSF accumulated, showed a 1% increase in the number of subchondral bone lesions with each HSF (IRR = 1.01; 95% CI 1.00–1.01). There was also a significant association between the total number of high‐speed works and the total number of MCP/MTP subchondral bone lesions (*p* < 0.001). For every high‐speed work accumulated, there was a 4% increase in the number of subchondral bone lesions (IRR = 1.04; 95% CI 1.02–1.05). When adjusted for time, there was not a significant association between the average speed per HSF and the total number of MCP/MTP subchondral bone lesions (IRR = 0.012; *p* = 0.2; 95% CI 0.30–1.23). Additionally, there was not a significant association between the amount of time off and the total number of subchondral bone lesions identified (IRR = 1.00; *p* = 0.8; 95% CI 0.98–1.02).

### 3.3 Effect of subchondral bone sclerosis and lesions on lameness

Lameness was not significantly associated with total subchondral bone sclerosis (IRR = 1.01; *p* = 0.3; 95% CI 0.99–1.02) or with total subchondral bone lesions (IRR = 1.07; *p* = 0.1; 95% CI 0.99–1.17).

### 3.4 Effect of subchondral bone sclerosis and lesions on joint effusion

Joint effusion was significantly associated with subchondral bone sclerosis such that there was a 1% increase in the likelihood of joint effusion with every 1 mm increase in sclerosis (IRR = 1.01; *p* = 0.02; 95% CI 1.00–1.02). Joint effusion was also significantly associated with subchondral bone lesions such that there was a 14% increase in the likelihood of effusion with each additional 1 subchondral bone lesion (IRR = 1.14; *p* < 0.01; 95% CI 1.04–1.25).

## 4 DISCUSSION

In this study, the association of high‐speed work with changes in the subchondral bone of the MCP/MTP joints, including the development of subchondral bone sclerosis and subchondral bone lesions, in Thoroughbred racehorses in their first year of training was examined using standing CBCT. The association of subchondral bone sclerosis and subchondral bone lesions with clinical parameters, including lameness and joint effusion, was also examined. The amount of subchondral bone sclerosis and the number of subchondral bone lesions were significantly associated with the number of HSFs trained and the number of high‐speed works. In contrast, the amount of subchondral bone sclerosis and the number of subchondral bone lesions were not associated with the average speed of HSFs and the number of rest weeks. Upon examination of the clinical parameters of lameness and joint effusion, joint effusion was significantly associated with subchondral bone sclerosis and subchondral bone lesions, but lameness was not.

Subchondral bone sclerosis, as estimated by hyperdensity on CBCT images, increased with the number of HSFs trained. Bone sclerosis, or increased bone volume fraction (BVF), is an adaptive process that occurs in response to cyclic loading of bone.^19^ The change in material properties of bone that occurs with increased BVF increases the fatigue life of bone.^19^, ^20^ Since bone adapts to the strain it experiences, exercise stimulates increases in BVF that are directly related to the speed at which the exercise occurs.^5^, ^21^, ^22^ However, this adaptive relationship is not infinite, leading to uncoupling of bone modelling and remodelling, with stress‐induced demineralisation or osteonecrosis a concern in racehorses in high‐speed training. In addition to increasing sclerosis, we found that there was an accumulation of total subchondral bone lesions over time and that the number of subchondral bone lesions was also significantly associated with the number of HSFs that the horse had accrued. Several studies have demonstrated the effect of exercise on bone, and it is now well accepted that while excessive training at high speed increases the risk of fatal musculoskeletal injuries,^7^, ^9^ short distances of high‐speed work are necessary for adaptive modelling and remodelling and increased fatigue life of bone.^23^, ^24^ For example, Parkin et al. found that 4 furlongs of high‐speed exercise per week may be all that is needed to promote healthy modelling and remodelling of bone.^15^ Verheyen et al. also showed that in a cohort of Thoroughbred racehorses, accumulation of canter exercise increased risk of fracture, while shorter periods of gallop exercise had a protective effect. However, the protective effect was negated by increasing distances in short periods, further supporting the delicate balance between bone adaptation and microdamage.^24^ While the potential negative effect of excessive high‐speed exercise on Thoroughbred training is understood, ideal training regimens that promote bone health and strength remain difficult to define.

While lameness was not associated with subchondral bone sclerosis or lesions in this study, joint effusion score was significantly associated with both. This was an interesting finding that could be due to the fact that subchondral bone lesions, while present on CT, are not well correlated with bone pain or lameness. Several recent studies have attempted to shed more light on the clinical relevance of CT findings in the equine fetlock. A recent study by Boros et al. found that hypoattenuating lesions of the sagittal ridge of MC3 noted on fan‐beam CT were not associated with lameness in a group of Thoroughbred racehorses.^12^ In contrast, Johnston et al. found that subchondral bone lesions in the parasagittal groove of MC3/MT3 were more prevalent in horses with fractures suggesting that hypodensities are consistent with microdamage accumulation^25^ and Brounts et al. found excellent correlation between lameness localised to the fetlock and abnormal CT imaging findings.^26^ In our study, in which horses tended to have multiple fetlocks with subchondral bone pathology, we must also consider that horses could have had multi‐limb lameness making lameness detection in a single limb difficult. Additionally, we did not perform diagnostic analgesia; therefore, it is also possible that there were other sources of lameness outside of the fetlock joint. In order to further examine the clinical impact of subchondral bone pathology noted on CT, localisation of lameness to joints with CT pathology would be necessary. Furthermore, additional tests such as contrast arthrography could be used to assess the joint for disruption of articular cartilage at areas of subchondral bone pathology, as has been demonstrated in several other studies.^25^, ^27^, ^28^ Joint effusion, as a measure of articular inflammation, could be an early indicator of changes in subchondral bone health and may be more easily detected in individual limbs than lameness. Further examination of the association between lameness and joint effusion, and MCP/MTP bone pathology is indicated.

Several studies have demonstrated the utility of both fan‐beam and cone‐beam CT for imaging the fetlocks of standing horses. While fan‐beam CT has greater contrast resolution and less susceptibility to motion artefact when compared with cone‐beam CT, both have been shown to have similar bone lesion detection capabilities.^10^, ^29^ In general, CT has also been shown to be superior to radiography and MRI for detection of most bone lesions.^11^, ^25^, ^26^, ^30^ Longitudinal imaging of the equine fetlock to assess the effects of exercise will likely benefit from the addition of functional imaging such as positron emission tomography (PET).^31^

This study had significant limitations, mainly related to variability in horse training and husbandry, that need to be considered. Horses that entered the study at 0 months were all in their 2‐year‐old year and at the beginning of high‐speed training; however, there was some variability in the amount of training horses underwent as yearlings prior to study enrolment. During the study period, horses were allowed to continue training and resting as prescribed by the individual trainer. Additionally, attrition due to retirement, change in geographic location, and change in ownership meant that not all 41 horses that entered the study were able to be imaged at all 3 time points. Due to the limitations of the CBCT system, we were not able to directly measure BMD and instead relied on hyperdensity (sclerosis) as an estimation of BMD. In addition, measurements of hyperdensity (sclerosis) were performed once by a single observer; therefore, no assessment of repeatability was performed. Due to the subjectivity in the assessment of the extent of hyperdensity, it is possible that both intra‐ and inter‐observer variability would have been noted. Total HSFs and total high‐speed works were only captured when horses completed an official clocked work or when horses raced, which limits the ability to assess the effect of other exercise on bone morphology and pathology. Finally, lesions associated with the sagittal ridge of MC3 were excluded from this study due to the high probability of these lesions being osteochondritis dissecans lesions as opposed to subchondral bone pathology occurring secondary to high‐speed work.^12^

In conclusion, this study provides further information that the amount of high‐speed work undertaken by Thoroughbred racehorses affects bone modelling and remodelling and the development of subchondral bone pathology and that overt lameness may be an insensitive criterion for the identification of at‐risk horses. Further research is needed to better determine the amount and frequency of high‐speed work that promotes bone health and limits stress‐induced injury. Looking forward, the use of wearable accelerometer‐based inertial measurement unit (IMU) sensors to more accurately document the number of loading cycles an individual horse experiences and monitoring for changes in horse speed and stride characteristics, in combination with the use of cross‐sectional imaging to monitor changes in bone morphology and the development of pathology, may provide owners, trainers and veterinarians the necessary information to prevent catastrophic bone injury.

## FUNDING INFORMATION

This study was supported by the Grayson‐Jockey Club Research Foundation.

## CONFLICT OF INTEREST STATEMENT

The authors declare no conflicts of interest.

## AUTHOR CONTRIBUTIONS

**Sarah A. Ciamillo:** Investigation; writing – original draft; writing – review and editing; data curation. **Kathryn W. Bills:** Conceptualization; investigation; funding acquisition; writing – review and editing; methodology. **Taryn M. Gassert:** Conceptualization; writing – review and editing. **Dean W. Richardson:** Conceptualization; funding acquisition; writing – review and editing. **Kara A. Brown:** Conceptualization; investigation; funding acquisition; writing – review and editing. **Darko Stefanovski:** Formal analysis; writing – original draft; writing – review and editing. **Kyla F. Ortved:** Conceptualization; investigation; funding acquisition; writing – original draft; methodology; validation; writing – review and editing; formal analysis; project administration; data curation; supervision; resources.

## DATA INTEGRITY STATEMENT

Kyla F. Ortved had access to all data in the study and takes responsibility for the integrity of the data and the accuracy of the data analysis.

## ETHICAL ANIMAL RESEARCH

This study was approved the University of Pennsylvania's Institutional Animal Care and Use Committee (IACUC) (#806710).

## INFORMED CONSENT

Owners consented to inclusion of animals in this study.

## Data Availability

The data that support the findings of this study are openly available in OSF at https://doi.org/10.17605/OSF.IO/UY7WF.
